# Repositioning Primary Health Care in Sierra Leone: From Geographic Access to Effective Coverage

**DOI:** 10.5334/aogh.5338

**Published:** 2026-08-17

**Authors:** Morie Vandi, John M. Koroma, Mustapha S. Kabba

**Affiliations:** 1 Ministry of Health, Freetown, Western Area Urban, Sierra Leone; 2 Medical University of South Carolina, Charleston, South Carolina, USA

**Keywords:** primary health care, universal health coverage, Sierra Leone, effective coverage, health financing, human resources for health, health system strengthening, quality of care

## Abstract

Primary health care (PHC) is central to Sierra Leone’s efforts to achieve universal health coverage. Despite substantial expansion of the peripheral health unit network, improvements in geographic access have not consistently translated into better health outcomes. This viewpoint argues that Sierra Leone must shift its focus from physical access to effective coverage, defined as the proportion of people who receive care of sufficient quality to produce meaningful health gains. Key challenges include persistent out-of-pocket expenditure, shortages of salaried health workers, weak service readiness, fragmented vertical programmes, limited digital infrastructure, and variable implementation capacity. Addressing these constraints will require reforms in financing, workforce formalisation, quality improvement, programme integration, and accountability. Strengthening these areas will be essential for ensuring that PHC delivers equitable and measurable health gains for all Sierra Leoneans.

## 1. Introduction

Primary health care (PHC) has been consistently positioned as the cornerstone of Sierra Leone’s health system and the primary pathway towards achieving universal health coverage (UHC). This policy orientation is reflected in national frameworks such as the *National Health Sector Strategic Plan 2021–2025* and the *National Health Compact 2025–2030*, both of which emphasise PHC as the first point of contact for the majority of the population and a central platform for delivering promotive, preventive, curative, and rehabilitative services across the life course [1, 2]. Despite this strong policy alignment, the translation of PHC investments into consistent and equitable health outcomes remains uneven, reflecting persistent structural and operational constraints.

Sierra Leone has made measurable progress in expanding geographic access to health services. Community health worker mapping data indicate that approximately 91% of the population lives within 5 km of a peripheral health unit (PHU), with community health workers extending geographic reach by a further 10% beyond fixed facility coverage [3]. This network expansion has contributed to improvements in key indicators, including a measurable reduction in maternal mortality over the past two decades [4, 5]. However, geographic access alone is insufficient to drive meaningful health gains. The more critical challenge is achieving *effective coverage*, defined as the fraction of potential health gain from an intervention that is actually received by those who need it [6, 7]. In Sierra Leone, like other low- and middle-income countries (LMICs), effective coverage remains constrained by fragmented care pathways, weak referral systems, inconsistent clinical quality, and limited continuity of care across levels of the health system [8, 9].

**Figure 1:**
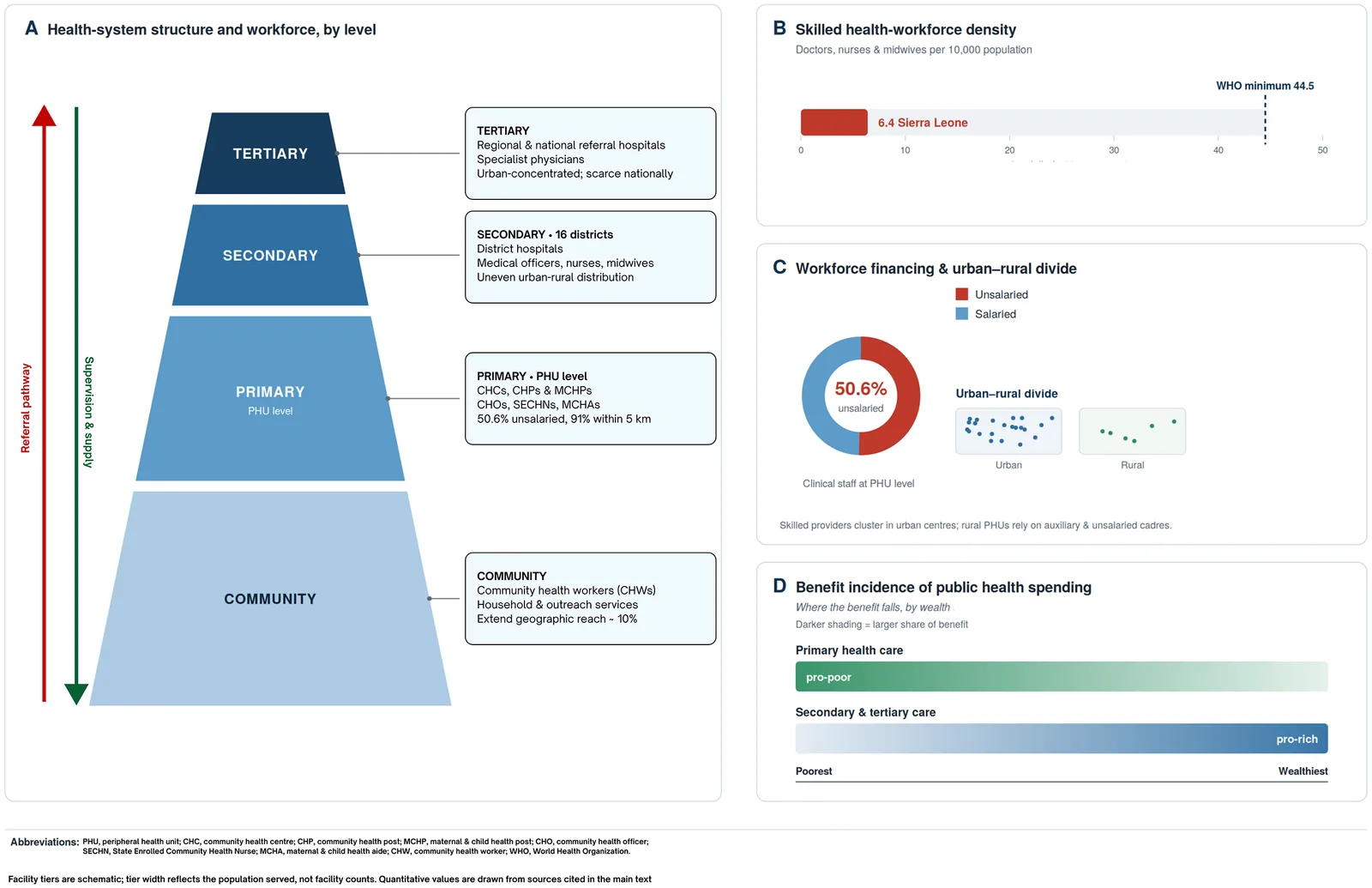
Structure of the Sierra Leone health system and the distribution and financing of the health workforce. **(A)** Service delivery structure from the community and peripheral health unit (PHU) levels through secondary (district hospital) and tertiary (regional and national referral hospital) levels, illustrating referral pathways, supervision and supply chains, and the distribution of key health workforce cadres. **(B)** Density of skilled health workers (doctors, nurses, and midwives) in Sierra Leone (6.4 per 10,000 population) compared with the WHO minimum threshold of 44.5 per 10,000 population. **(C)** Distribution of salaried and unsalaried clinical staff at PHU level, showing that 50.6% of clinical staff are unsalaried and that unsalaried staff outnumber salaried staff in 7 of the 10 districts surveyed. **(D)** Schematic illustration of the distribution of public health spending, showing that primary health care expenditure is relatively pro-poor whereas secondary and tertiary care expenditure is relatively pro-rich. Panel D illustrates the direction of benefit incidence only and does not represent budget shares. The figure is an original synthesis based on data from the Sierra Leone Human Resources for Health Country Profile and published studies on the health workforce and health financing [3, 11–13].

To structure this analysis, we draw on the World Health Organization (WHO) health systems framework, which characterises a health system in terms of six interdependent building blocks: health financing; the health workforce; service delivery; access to essential medicines, vaccines, and health technologies; health information systems; and leadership and governance [10]. Figure 1 depicts the structure of Sierra Leone’s health system, from the community and PHU levels through district and tertiary referral hospitals, together with the workforce constraints that affect each level. In the sections that follow, we examine each building block in turn, considering how it currently constrains, or could enable the transition from geographic access to effective coverage, and we identify the reforms most likely to close that gap.

## 2. Health Financing and Financial Protection

Financing remains a central bottleneck in the delivery of effective PHC. Although Sierra Leone launched the Free Health Care Initiative (FHCI) in 2010 to abolish user fees for pregnant women, lactating mothers, and children under five [14], out-of-pocket (OOP) expenditure continues to represent a substantial share of total health spending. Based on WHO Global Health Expenditure Database estimates, OOP expenditure accounted for approximately 50% of current health expenditure as of 2021, one of the highest burdens in the sub-region [15, 16]. Despite formal fee abolition under the FHCI, OOP expenditure has persisted among eligible beneficiaries. Van Duinen et al. [17] found that among 1,146 women who underwent caesarean section across 9 hospitals in Sierra Leone, the median expenditure was 23 United States Dollars (USD), and 12% experienced catastrophic expenditure, indicating that the FHCI provides only partial financial protection for the most vulnerable [17].

High OOP costs are associated with delayed care-seeking, incomplete treatment, and reduced adherence, particularly among vulnerable populations [18]. Addressing these challenges requires a shift from nominal service entitlements towards more robust financing mechanisms, including strategic purchasing, direct facility financing, and strengthened accountability systems to reduce informal payments and ensure affordability at the point of care. Advancing social health insurance reforms currently under consideration by the Government of Sierra Leone will be essential to achieving durable financial protection.

## 3. Health Workforce (Human Resources for Health)

Human resources for health represent another critical constraint. Sierra Leone faces a severe shortage of skilled health workers, with significant urban–rural disparities in workforce distribution. The density of doctors, nurses, and midwives was estimated at 6.4 per 10,000 population, well below the WHO-recommended minimum threshold of 44.5 per 10,000 required to make meaningful progress towards UHC [11, 19]. A mixed-methods survey across 10 of Sierra Leone’s 16 districts, conducted in 2023–2024, found that just over half (50.6%) of clinical staff at PHUs were unsalaried, operating outside formal payroll systems with limited accountability [19]. In 7 of the 10 districts surveyed, unsalaried staff outnumbered salaried counterparts [19].

This structural reliance on unpaid auxiliary cadres, including State Enrolled Community Health Nurses (SECHNs) and Maternal and Child Health Aides (MCHAs), undermines service continuity, quality, and accountability. A growing pool of qualified but unsalaried graduates, combined with the limited fiscal capacity to absorb them onto the government payroll, creates a systemic tension that threatens the long-term sustainability of the workforce [12]. Strengthening this workforce requires formal integration into the health system, standardised training and scopes of practice, supportive supervision, and sustainable remuneration structures, particularly in rural and underserved areas.

## 4. Service Delivery: Service Readiness and Quality of Care

Service readiness, which is the availability of the trained staff, equipment, essential medicines, diagnostics, and infrastructure required to deliver services, and quality of care remain fundamental determinants of PHC performance. A cross-sectional facility survey in Kono District found that the most significant barrier to service readiness was essential medicines availability, with facilities reporting a mean readiness score of only 32% on the medicines domain [20]. A nationally representative infrastructure mapping study of 72 health facilities found that 82% lacked institutionally provided internet, and 40% of Maternal and Child Health Posts had no electricity source [21]. The 2018 World Bank Service Delivery Indicators survey, conducted across a nationally representative sample of health facilities throughout Sierra Leone, documented deficiencies in equipment availability, provider absenteeism, and diagnostic accuracy [22].

These constraints directly affect the safety, effectiveness, and acceptability of care at the frontline. Quality of care must therefore be repositioned as a central objective of PHC reform, encompassing care that is effective, safe, and people-centred [23]. Without addressing these dimensions, improvements in access are unlikely to translate into sustained gains in health outcomes.

## 5. Access to Essential Medicines, Vaccines, and Health Technologies

Reliable access to quality-assured essential medicines, vaccines, and health technologies is a distinct building block of the health system and a precondition for effective coverage; where commodities, cold-chain capacity, or diagnostics are lacking, even a well-staffed facility cannot deliver effective care. Sierra Leone has demonstrated notable success in implementing vertical health programmes, particularly in immunisation and malaria control. The introduction of the RTS,S/AS01 malaria vaccine into the routine immunisation schedule in December 2024, making Sierra Leone the third country globally to do so, represents a significant milestone in the country’s public health trajectory [24]. However, the coexistence of strong vertical programmes alongside a relatively weak horizontal PHC system creates risks of fragmentation, supply chain duplication, and parallel data systems. To maximise impact, vertical interventions must be embedded within a strengthened PHC platform that supports comprehensive, continuous, and coordinated care across the life course. This requires deliberate alignment of workforce planning, supply chains, financing, and health information systems to avoid inefficiencies and ensure coherence at the district and facility levels [25, 26].

## 6. Health Information Systems and Digital Health

Digital health initiatives are increasingly being recognised as enablers of PHC strengthening. The *Digital Health Roadmap 2024-2026* outlines efforts to improve health information systems, surveillance, and data use for decision-making [27]. When effectively implemented, digital tools can support real-time monitoring of service delivery, enhance supply chain visibility, improve referral coordination, and enable data-driven management at facility and district levels. However, the infrastructure gap is significant: only 10 of 72 surveyed health facilities had functional official internet connectivity, and 43% identified inadequate electricity as the primary threat to digitisation [21]. Fragmented or parallel digital systems risk increasing administrative burden without improving system performance.

## 7. Leadership and Governance

A critical, yet often underemphasised, determinant of PHC performance is governance and implementation capacity at the district and facility levels. While national policies are generally well-articulated, execution varies significantly across contexts. Effective PHC delivery depends on strong district health management teams, functional supervision systems, routine performance monitoring, and the use of data to inform local decision-making [28]. Research on the redistributive effects of public health spending in Sierra Leone has demonstrated that while PHC benefits are pro-poor, secondary and tertiary benefits disproportionately favour higher-income quintiles, and when overall benefits are adjusted for need, the public health system remains inequitable [13]. Facility-level autonomy, coupled with clear accountability mechanisms, is essential for improving responsiveness and equity in service delivery. Ultimately, the gap between policy intent and service delivery outcomes reflects limitations in implementation fidelity rather than policy design.

## 8. Conclusion

Primary health care in Sierra Leone has evolved into a central pillar of health system reform, with strong policy backing and increasing political commitment to UHC. However, the next phase of reform must move decisively beyond expanding geographic access towards ensuring effective coverage. This requires reducing financial barriers, formalising and professionalising the health workforce, including committing sufficient recurrent budget to absorb trained but currently unsalaried health workers onto the government payroll, improving service readiness and quality, securing a reliable supply of essential medicines, vaccines, and health technologies, strengthening health information systems, and reinforcing leadership, governance, and accountability at all levels. If these priorities are addressed with sustained commitment and implementation fidelity, PHC has the potential to deliver substantial and equitable health gains for all Sierra Leoneans. Without such efforts, the country risks maintaining a system characterised by broad geographic reach but limited health impact.

## Data Availability

This is a commentary and does not generate primary data. All sources cited are publicly available and referenced accordingly.

## References

[r1] Government of Sierra Leone. National Health Compact. Government of Sierra Leone; 2025. https://thedocs.worldbank.org/en/doc/0273f33ab6ee48c5d842108b9b55c789-0140022025/related/National-Health-Compact-Sierra-Leone.pdf.

[r2] Ministry of Health. Sierra Leone National Multi-Sectoral Strategy to Prevent and Control Anaemia 2018–2025. Published 2018.

[r3] Directorate of Human Resources for Health, Ministry of Health and Sanitation SL. Human Resources for Health Country Profile: Sierra Leone. Government of Sierra Leone; 2016. https://www.afro.who.int/sites/default/files/2017-05/hrhprofile16.pdf.

[r4] United Nations Population Fund. Sierra Leone’s maternal mortality ratio declines: UNFPA launches midwifery accelerator to tackle remaining challenges. Published 2025. https://sierraleone.unfpa.org/en/news/sierra-leones-maternal-mortality-ratio-declines-unfpa-launches-midwifery-accelerator-tackle.

[r5] World Health Organization. WHO Sierra Leone Annual Report 2022. WHO Regional Office for Africa; 2022. https://www.afro.who.int/sites/default/files/2023-07/WHO%20Sierra%20Leone%20Annual%20Report%20for%202022.pdf.

[r6] Shengelia B, Tandon A, Adams OB, Murray CJL. Access, utilization, quality, and effective coverage: An integrated conceptual framework and measurement strategy. Soc Sci Med. 2005;61(1):97–109. doi:10.1016/j.socscimed.2004.11.055. 15847965

[r7] Tanahashi T. Health service coverage and its evaluation. Bull World Health Organ. 1978;56(2):295. https://iris.who.int/handle/10665/261736. 96953 PMC2395571

[r8] Amouzou A, Leslie HH, Ram M, et al. Advances in the measurement of coverage for RMNCH and nutrition: From contact to effective coverage. BMJ Glob Health. 2019;4(Suppl 4):e001297. doi:10.1136/bmjgh-2018-001297. PMC6590972 31297252

[r9] Victora C, Vaughan JP, Lombardi C, et al. Evidence for protection by breast-feeding against infant deaths from infectious diseases in Brazil. Lancet. 1987;330(8554):319–322. doi:10.1016/s0140-6736(87)90902-0. 2886775

[r10] World Health Organization. Everybody’s Business: Strengthening Health Systems to Improve Health Outcomes: WHO’s Framework for Action. World Health Organization; 2007. https://apps.who.int/iris/handle/10665/43918.

[r11] Pieterse P, Saracini F. Unsalaried health workers in Sierra Leone: A scoping review of the literature to establish their impact on healthcare delivery. Int J Equity Health. 2023;22(1):255. doi:10.1186/s12939-023-02066-3. 38066622 PMC10709924

[r12] Pieterse P, Saracini F. Improving Sierra Leone’s skilled health-worker-to-population ratio: How unsalaried and auxiliary health workers are barriers in its path to universal health coverage. BMJ Glob Health. 2025;10(11):e021043. doi:10.1136/bmjgh-2025-021043. PMC12636922 41253432

[r13] Gabani J, Mazumdar S, Hadji SB, Amara MM. The redistributive effect of the public health system: The case of Sierra Leone. Health Policy Plan. 2024;39(1):4–21. doi:10.1093/heapol/czad100. 37990623 PMC10775248

[r14] Witter S, Brikci N, Harris T, et al. The free healthcare initiative in Sierra Leone: Evaluating a health system reform, 2010–2015. Int J Health Plann Manage. 2018;33(2):434–448. doi:10.1002/hpm.2484. 29327367

[r15] Ministry of Health and Sanitation SL. Enhancing Investments in Primary Health Care for the Achievement of Universal Health Coverage in Sierra Leone. Government of Sierra Leone; 2025. https://chwcentral.org/wp-content/uploads/Enhancing-Investments-in-Primary-Health-Care-for-the-Achievement-of-Universal-Health-Coverage-in-Sierra-Leone.pdf.

[r16] World Bank. Out-of-pocket expenditure (% of current health expenditure) – Sierra Leone. World Development Indicators. Published 2025. https://data.worldbank.org/indicator/SH.XPD.OOPC.CH.ZS?locations=SL.

[r17] van Duinen AJ, Westendorp J, Ashley T, et al. Catastrophic expenditure and impoverishment after caesarean section in Sierra Leone: An evaluation of the free health care initiative. PLoS One. 2021;16(10):e0258532. doi:10.1371/journal.pone.0258532. 34653191 PMC8519447

[r18] Edoka I, Ensor T, McPake B, Amara R, Tseng FM, Edem-Hotah J. Free health care for under-fives, expectant and recent mothers? Evaluating the impact of Sierra Leone’s free health care initiative. Health Econ Rev. 2016;6(1):19. doi:10.1186/s13561-016-0096-4. 27215909 PMC4877339

[r19] Pieterse P, Saracini F. Employed but unpaid, volunteers or paradoxical surplus? Sierra Leone’s unsalaried health workforce. Int J Health Plann Manage. 2026;41(1):7–16. doi:10.1002/hpm.70016. 40778790 PMC12794118

[r20] Higgins J, Jerome JG, Boima F, et al. Community and facility-level barriers to achieving UHC in Kono District, Sierra Leone and Maryland County, Liberia. PLOS Glob Public Health. 2023;3(6):e0002045. doi:10.1371/journal.pgph.0002045. 37363882 PMC10292700

[r21] Chukwu E, Garg L, Foday E, Konomanyi A, Wright R, Smart F. Electricity, computing hardware, and internet infrastructures in health facilities in Sierra Leone: Field mapping study. JMIR Med Inform. 2022;10(2):e30040. doi:10.2196/30040. 35113026 PMC8855303

[r22] Statistics Sierra Leone, World Bank. Service Delivery Indicators Health Survey 2018 – Sierra Leone. World Bank Microdata Library. Published 2018. doi:10.48529/73vd-qp74.

[r23] OECD, World Health Organization, World Bank Group. Delivering Quality Health Services: A Global Imperative for Universal Health Coverage. World Health Organization; 2018. doi:10.1787/9789264300309-en.

[r24] Kangbai DM, Sesay EN, Clemens-Kangbai N, et al. Introduction and roll-out of malaria vaccine in Sierra Leone: Early lessons and experiences from the first 8 months of implementation. Lancet Prim Care. 2025;1(4):100037. doi:10.1016/j.lanprc.2025.100037.

[r25] Atun R, de Jongh T, Secci F, Ohiri K, Adeyi O. Integration of targeted health interventions into health systems: A conceptual framework for analysis. Health Policy Plan. 2010;25(2):104–111. doi:10.1093/heapol/czp055. 19917651

[r26] World Health Organization. The World Health Report 2008: Primary Health Care – Now More Than Ever. World Health Organization; 2008. https://www.paho.org/sites/default/files/PHC_The_World_Health_Report-2008.pdf.

[r27] Ministry of Health SL. National Digital Health Roadmap 2024–2026. Government of Sierra Leone; 2024. https://mohs.gov.sl.

[r28] World Health Organization, United Nations Children’s Fund (UNICEF). Operational Framework for Primary Health Care: Transforming Vision into Action. World Health Organization; 2020. https://www.who.int/publications/i/item/9789240017832.

